# *Cuscuta arvensis* Beyr “Dodder”: *In Vivo* Hepatoprotective Effects Against Acetaminophen-Induced Hepatotoxicity in Rats

**DOI:** 10.1089/jmf.2017.0139

**Published:** 2018-06-01

**Authors:** Ufuk Koca-Caliskan, Ismet Yilmaz, Asli Taslidere, Funda N. Yalcin, Ceylan Aka, Nazim Sekeroglu

**Affiliations:** ^1^ Department of Pharmacognosy, Faculty of Pharmacy, Gazi University, Etiler-Ankara, Turkey.; ^2^ Department of Pharmacology, Faculty of Pharmacy, Inonu University, Malatya, Turkey.; ^3^ Department of Histology-Embryology, Faculty of Medicine, Inonu University, Malatya, Turkey.; ^4^ Department of Pharmacognosy, Faculty of Pharmacy, Hacettepe University, Altindag-Ankara, Turkey.; ^5^ Department of Biology, Faculty of Art and Science, Kilis 7 Aralik University, Kilis, Turkey.

**Keywords:** *acetaminophen*, *Cuscuta*, *hepatoprotective*, *histopathology*, in vivo *antioxidant activity*

## Abstract

*Cuscuta arvensis* Beyr. is a parasitic plant, and commonly known as “dodder” in Europe, in the United States, and “tu si zi shu” in China. It is one of the preferred spices used in sweet and savory dishes. Also, it is used as a folk medicine for the treatment particularly of liver problems, knee pains, and physiological hepatitis, which occur notably in newborns and their mothers in the southeastern part of Turkey. The purpose of this study was to investigate the hepatoprotective effects and antioxidant activities of aqueous and methanolic extracts of *C. arvensis* Beyr. on acetaminophen (APAP)-induced acute hepatotoxicity in rats. The results were supported by subsequent histopathological studies. The hepatoprotective activity of both the aqueous and methanolic extracts at an oral dose of 125 and 250 mg/kg was investigated by observing the reduction levels or the activity of alkaline phosphatase, alkaline transaminase, aspartate aminotransferase, blood urine nitrogen, and total bilirubin content. *In vivo* antioxidant activity was determined by analyzing the serum superoxide dismutase, malondialdehyde, glutathione, and catalase levels. Chromatographic methods were used to isolate biologically active compounds from the extract, and spectroscopic methods were used for structure elucidation. Both the methanolic and aqueous extracts exerted noticable hepatoprotective and antioxidant effects supporting the folkloric usage of dodder. One of the bioactive compounds was kaempferol-3-*O*-rhamnoside, isolated and identified from the methanolic extract.

## Introduction

Hepatotoxicity is a leading cause of lack of continuation of a drug therapy. Research on hepatotoxicity suggests that reactive metabolites or xenobiotics, excessive alcohol consumption, environmental, and some disease conditions are responsible for moderate to severe liver injury. Compounds causing hepatotoxicity directly affect the mitochondrial respiratory chain, also, the permeability transitional pore, cytochromes P-450, glutathione (GSH) S-acyltransferases, and other antioxidant mechanisms in living organisms.^1^

Several plant extracts and polyherbal medications have been clinically approved for their effective hepatoprotective activity.^2^ Dodder has been consumed as a herbal tea and medicinal plant in different parts of the world. Antioxidant activity of dodder, which is used as a Turkish folk medicine to alleviate jaundice in newborn infants, suggests that it may be pharmacologically effective on the liver and against liver toxicity. Pharmacological studies have revealed that *Cuscuta* sp. (Convolvulaceae) have antiaging, anticancer, antihypertensive, anti-inflammatory, antiosteoporotic, antioxidant, hepatoprotective, immunomodulatory, immunostimulant, and memory enhancing activities.^3–13^ Although the phytochemical content of *Cuscuta* sp. differs according to plant variation, to date, flavonoids, polysaccharides, alkaloids, and lignans have been isolated from different *Cuscuta* sp.^14^ However, there appears to be no phytochemical or biological activity studies reported on *Cuscuta arvensis* Beyr. until now.

In this study, both aqueous and methanolic extracts of *C. arvensis* were applied to acute hepatotoxicity-induced animals dosed with acetaminophen. Some biochemical parameters (aspartate aminotransferase [AST], alkaline phosphatase [ALP], alkaline transaminase [ALT], blood urine nitrogen [BUN], and total bilirubin [TB]) and liver tissues of the rats were analyzed with histopathological methods to observe the hepatoprotective effects of the extracts on the liver tissue. This work then led to phytochemical, chromatographic, and spectral methods to isolate and identify the major and effective compound/s of the extract.

## Materials and Methods

### Chemicals

Acetaminophen was purchased from Sigma Aldrich (St. Louis, MO, USA). Solvents were obtained from Merck in HPLC grade.

### Plant material and preparation of the extracts

The whole flowering plant materials were collected during May 2011 at the Mardin region of Turkey. A prepared herbarium specimen was identified by Prof. Nazim Sekeroglu and the specimens (KHB-78) were deposited at the Department of Biology in Kilis 7 Aralik University, Turkey.

The dodder plant samples were powdered and divided into 400 g samples, which were then extracted separately with either methanol (4 × 1000 mL) or water (4 × 1000 mL). The methanolic extract was evaporated until dryness, whereas the aqueous extract was evaporated first, then freeze dried in a lyophilizer. The yields of methanolic extract and aqueous extract were 14.95% and 8.50%, respectively.

### Test animals

Seventy Sprague–Dawley female rats (247 ± 15.3 g) were purchased from the Experimental Animal Research and Production Center of Inonu University, (Malatya, Turkey). They were housed on a 12 h light–dark cycle at 21°C ± 2°C, and in a relative humidity of 53% ± 3%. Animals were fed on standard diet pellets (normal chow) and water (tap water). The animal study protocol (No. 2011/A-94) was approved by the Ethical Committee, Faculty of Medicine, Inonu University.^2^ The rats were divided into six groups, as follows:

Group I (CONTROL) was fed with normal chow and tap water *ad libitum* (*n* = 7).
Group II (APAP) received a 850 mg/kg single acute toxic dose acetaminophen solution given by gastric gavage (*n* = 7).
Group III received 850 mg/kg single dose acetaminophen, and in addition either 125 mg/kg methanolic or aqueous extract of dodder solution was given by gastric gavage for 6 days (*n* = 7).
Group IV was given 850 mg/kg single dose acetaminophen, in addition 250 mg/kg methanolic or aqueous extract of dodder solution was given by gastric gavage for 6 days (*n* = 7).
Group V was given 125 mg/kg methanolic or aqueous extract of dodder solution by gastric gavage for 7 days (*n* = 7).
Group VI was given 250 mg/kg methanolic or aqueous extract of dodder solution by gastric gavage for 7 days (*n* = 7).

During the study periods, no toxic or adverse effects or death were observed in any of the animals.

### Measurement of liver functions

After day 7, the animals were sacrificed under kethamine+xylazine anesthesia. Blood, liver, and renal tissue samples were taken from all the animals. Blood samples were centrifuged at 3000 *g* for 10 min. to obtain serum samples that were stored at −20°C until analyzed. Liver tissue was immediately washed with saline and frozen at −40°C until needed for the study. Liver TB and other liver function markers AST, ALT, and ALP were measured (Table 1). *In vivo* antioxidant activity was determined by measuring and analyzing the serum superoxide dismutase (SOD), catalase (CAT), glutathione peroxidase (GPx), and malondialdehyde (MDA) by Abbott clinical autoanalyzer (Abbott Diagnostics; Abbott Park, IL, USA) using an ion-selective electrode method (Architect c16000).

**Table 1.** Results of Blood Urea Nitrogen, Total Bilirubin, Aspartate Aminotransferase, Alkaline Transaminase, and Alkaline Phosphatase Parameters of the Control and Only APAP, APAP+Extracts (APAP+Aq, APAP+Cus), and only Extracts (Cus-Aq, Cus-MeOH) Given Rat Groups

	*Parameters*				
*Groups*	*BUN (mg/dL)*	*TB (mg/dL)*	*AST (U/L)*	*ALT(U/L)*	*ALP (U/L)*
Control	19.4 ± 2.4 (16–24)	0.7 ± 0.4	188 ± 67.5 (89–266)	67 ± 22.1 (42–99)	190 ± 54.9 (128–296)
APAP	19.9 ± 2.3 (16–22)	0.8 ± 0.3	232 ± 57.2 (175–329)	113 ± 28.2 (85–169)	201 ± 29.4 (172–243)
APAP +125-Aq	23.3 ± 2.1 (21–27)	0.6 ± 0.4	204 ± 100.9 (81–388)	116 ± 97.9 (35–320)	163 ± 73.2 (55–254)
APAP +250-Aq	21.4 ± 3.6 (15–26)	0.6 ± 0.4	168 ± 60.5 (69–264)	94 ± 66.8 (30–236)	152 ± 43.7 (85–203)
APAP +125-MeOH	**17.9 ± 2.7 (14–22)^b^**	0.7 ± 0.4	113 ± 27.6 (76–161)	**42 ± 7.8 (33–55)^a^**	125 ± 39.6 (54–171)
APAP +250-MeOH	**17.6 ± 2.1 (15–20)^b^**	0.6 ± 0.4	141 ± 23.0 (116–168)	**48 ± 7.1 (35–56)^a^**	175 ± 41.6 (111–242)
CUST 125-Aq	19.0 ± 2.6 (16–22)	0.8 ± 0.3	174 ± 75.8 (109–307)	81 ± 52.2 (43–191)	185 ± 52.7 (117–274)
CUST 250-Aq	18.1 ± 2.9 (15–22)	0.9 ± 0.0	162 ± 54.7 (106–271)	57 ± 17.5 (27–74)	141 ± 28.8 (106–192)
CUST 125-MeOH	21.1 ± 1.5 (20–24)	0.5 ± 0.4	188 ± 96.1 (98–380)	69 ± 25.9 (40–120)	161 ± 40.5 (108–214)
CUST 250-MeOH	19.9 ± 2.9 (14–23)	0.3 ± 0.4	241 ± 88.2 (82–542)	77 ± 48.2 (31–170)	186 ± 62.9 (110–282)

^a,b^ Express the statistical significance of the values.

ALP, alkaline phosphatase; ALT, alkaline transaminase; AST, aspartate aminotransferase; BUN, blood urine nitrogen; TB, total bilirubin.

### Biochemical analysis

The samples were homogenized in ice-cold 0.1 M Tris–HCl buffer (pH 7.5) (containing protease inhibitor, phenylmethylsulfonyl fluoride, 1 mM) with a homogenizer at 11,448 *g* for 2 min at +(4–8)°C. The homogenates were used to measure the levels of MDA, GSH, SOD, and CAT.

### MDA and GSH assay

MDA, referred to as thiobarbituric acid reactive substances (TBARS), was measured with tiobarbituric acid at 535 and 520 nm in a spectrophotometer as previously described.^15^ Results were reported as nmol/g wet tissue. Reduced GSH concentrations in the homogenates were measured according to the previously described spectrophotometric method.^16^

### SOD assay

SOD activity was measured by determining the reduction of nitroblue tetrazolium by the superoxide anion produced with xanthine and xanthine oxidase.^17^ One unit of SOD was defined as the amount of protein that inhibits the rate of nitro blue tetrazolium (NBT) reduction by 50% and the results are reported as units per milligram (U/mg) protein. The specific activity of the enzymes is expressed in units per miligram protein. Proteins in the seminal fluid were determined by the method of Lowry *et al.*^18^ Peroxidase activity was measured by monitoring the oxidation of reduced nicotinamide–adenine dinucleotide phosphate at 340 nm, as described by Paglia and Valentine, and the results are reported as units per gram protein using an extinction coefficient of 6.22 × 10^−6^/M/cm.^19^

### Determination of CAT activity

CAT activity was measured according to Aebi's method, by determining the rate constant k (dimension: s−1,k) of H_2_O_2_ (initial concentration 10 mM) at 240 nm by the spectrophotometer. Activity was reported as k (constant rate) per gram (U/g) protein.^20^

### Statistical analysis

Statistical analysis was performed using SPSS 15 program. All data are reported as mean values ± standard deviations. A value of (*P* < .05) was considered as significant.

### Histological examination

Liver slices were fixed with 10% formalin in phosphate-buffered saline for 24 h and embedded in paraffin. Sections of 4 *μ*m thickness were stained with hematoxylin–eosin (H-E) and were analyzed under the microscope to observe histopathological changes in the liver. Pictures of each slide were taken at 100 × magnification.

For the light microscopical evaluation, liver samples were fixed in 10% formalin and were processed by routine tissue techniques, then they were embedded in paraffin. Paraffin-embedded specimens were cut into 5-mm thick sections, mounted on slides, and stained with H-E. Sections were examined under a Leica DFC280 light microscope combined with Leica Q Win Image Analysis System (Leica Micros Imaging Solutions Ltd., Cambridge, United Kingdom).

An overall score of liver damage severity was semiquantitively assessed as follows: eosinophilic stained and pyknotic nuclei cells, necrosis, hemorrhage, and mononuclear cell infiltration. The microscopic score of each tissue was calculated as the sum of the scores given to each criterion and scored as follows: 0, none; 1, mild; 2, moderate; 3, severe.

Statistical analyses were made using an SPSS 13 and MedCalc program. All groups were compared by the nonparametric Kruskal–Wallis test. Exact *P*-values were given where available, and *P* < .0001 was accepted as statistically significant. All results are expressed as means ± standard error.

### Isolation and determination of major/effective compound/s

The methanolic extract (40 g; yield: 20.67%) was dissolved in water (100 mL) and was first submitted to a polyamide column and eluted with a solvent gradient of MeOH–H_2_O (0:100 → 100:0) to afford nine main fractions (Frs. A–I, 200 mL each). All of the fractions were rich in sugars and flavonoids. The major flavonoid was isolated from fraction E (160 mg), using a Sephadex LH-20 column chromatography eluting with chloroform–methanol (1:1) to give six fractions (Frs. E_1–6_). Fr E_2_ was subjected to preparative TLC analysis for further purification to yield **1** (5 mg).

Structure elucidation of **1** was determined by NMR and mass spectrometry.^21–23^

## Results

### Measurement of liver functions

Plasma levels of BUN, TB, AST, ALT, and ALP were determined as measures of liver function in rats (Table 1). Only groups (CUST-MeOH, Cus-Aq) given extracts had similar results to the control group. The data have shown that only extract-given groups without any toxic drug neither had toxicity on kidney nor on liver (Table 1). Plasma levels of BUN significantly increased in the APAP intoxicated group, whereas in the Cuscuta extract groups the levels went back to the control (19.4 ± 2.4) levels. A similar pattern was observed with the TB levels, APAP (0.8 ± 0.3) caused a drastic increase, and with treatment of the Cuscuta extract with APAP or without APAP, the TB level went back to control levels (0.7 ± 0.4). Administration of APAP induced severe hepatic injury, as shown by increases in AST (232 ± 57.2), ALT (113 ± 28.2), and ALP (201 ± 29.4) enzyme levels (*P* < .01). Treatment with Cuscuta mostly with methanolic extracts of Cuscuta (APAP +125MeOH: AST 113 ± 27.6, ALT 42 ± 7.8, ALP 125 ± 39.6; APAP +250MeOH: AST 141 ± 23.0, ALT 48 ± 7.1, ALP 175 ± 41.6) significantly reduced plasma levels of those enzyme levels in a dose-dependent manner (*P* < .05 or .01). The recovery from APAP-induced toxicity produced by treatment with Cuscuta was similar to that seen in the control AST (188 ± 67.5), ALT (67 ± 22.1), and ALP (190 ± 54.9) (Table 1).

### Biochemical analysis

The lipid peroxidation was examined by determining MDA content in liver tissue homogenate. The MDA content in the APAP group (732.2 ± 96.6) was significantly higher than that in the control group (582.6 ± 132.4). In the Cuscuta-treated groups, mostly with the methanolic extract (APAP +250MeOH 553.3 ± 51.6), the MDA level decreased compared with the APAP group showing an indication of recovery from intoxication.

The levels of GSH, CAT, and SOD were determined in liver tissues. Constant levels of GSH components were found in the Cuscuta-treated groups (APAP +125MeOH: 993.7 ± 116.3: APAP +250MeOH: 1736.3 ± 165.9) compared with the APAP group (*P* < .01) (Table 2). Interestingly, APAP and aqueous Cuscuta-treated group (APAP +125Aq: 899.4 ± 34.6) and only aqueous Cuscuta (CUST 125Aq: 931.4 ± 82.5) given group have shown an increase in GSH levels. Cuscuta treatment significantly prevented inhibition of SOD and CAT activity caused by APAP toxicity compared with the APAP group (*P* < .01) (Table 2).

**Table 2.** Results of Serum Superoxide Dismutase, Malondialdehyde, Glutathione, Catalase, and Parameters of the Control, Only APAP, APAP+Extracts (APAP+Aq, APAP+Cus), and Only Extracts (Cus-Aq, Cus-MeOH) Given Rat Groups

	*Parameters*			
*Groups*	*SOD (U/g protein)*	*MDA (nmol/g)*	*GSH (nmol/g)*	*CAT (K/g protein)*
Control	52.9 ± 8.4 (43–646)	582.6 ± 132.4 (429–814)	1223.4 ± 134 (1026–1468)	34.3 ± 3.1 (30–37)
APAP	58.4 ± 13 (40–75)	732.4 ± 96.6 (429–814)	909.1 ± 158 (674–1088)^djf^	28.2 ± 4.7 (22–36)
APAP +125-Aq	23.9 ± 9.2 (17–36)^hfgi^	732.4 ± 96.6 (600–905)	899.4 ± 34.6 (846–955)^djf^	30.6 ± 4.6 (24–36)
APAP +250-Aq	51.1 ± 6.5 (43–60)	603 ± 92.2 (506–717)	1445.1 ± 255.8 (1154–1795)	31.7 ± 3.3 (27–37)
APAP +125-MeOH	46.7 ± 8.6 (34–60)	658.6 ± 81 (556–781)	993.7 ± 116.3 (827–1154)^f^	32 ± 3.1 (31–39)
APAP +250-MeOH	61.9 ± 18.7 (35–83)	553.3 ± 51.6 (502–626)	1736.3 ± 165.9 (1487–1897)^djf1^	34.7 ± 3.7 (31–39)
CUST 125-Aq	66.2 ± 12.7 (49–82)	712.0 ± 47.7 (636–775)	931.4 ± 82.5 (800–1053)^djf^	32.3 ± 2.5 (28–35)
CUST 250-Aq	59.2 ± 6.4 (53–70)	584.9 ± 44 (539–653)	1088 ± 61.3 (981–1167)	32.2 ± 3.7 (27–36)
CUST 125-MeOH	68.3 ± 8.3 (57–76)	540.8 ± 63.7 (432–620)^gc^	916.0 ± 69.4 (809–996)^djf^	31.4 ± 1.7 (30–35)
CUST 250-MeOH	52.4 ± 8.2 (42–62)	655.6 ± 85.9 (429–905)	1491.7 ± 213.4 (1205–1840)	29.6 ± 12.3 (2–38)

Kruskal–Wallis test was used to compare the groups. The results are tabled as showing both SD and (min.–max).

Superscript letters express statistical significance.

CAT, catalase; GSH, glutathione; MDA, malondialdehyde; SOD, superoxide dismutase.

### Histological examination

**FIG. 1.**
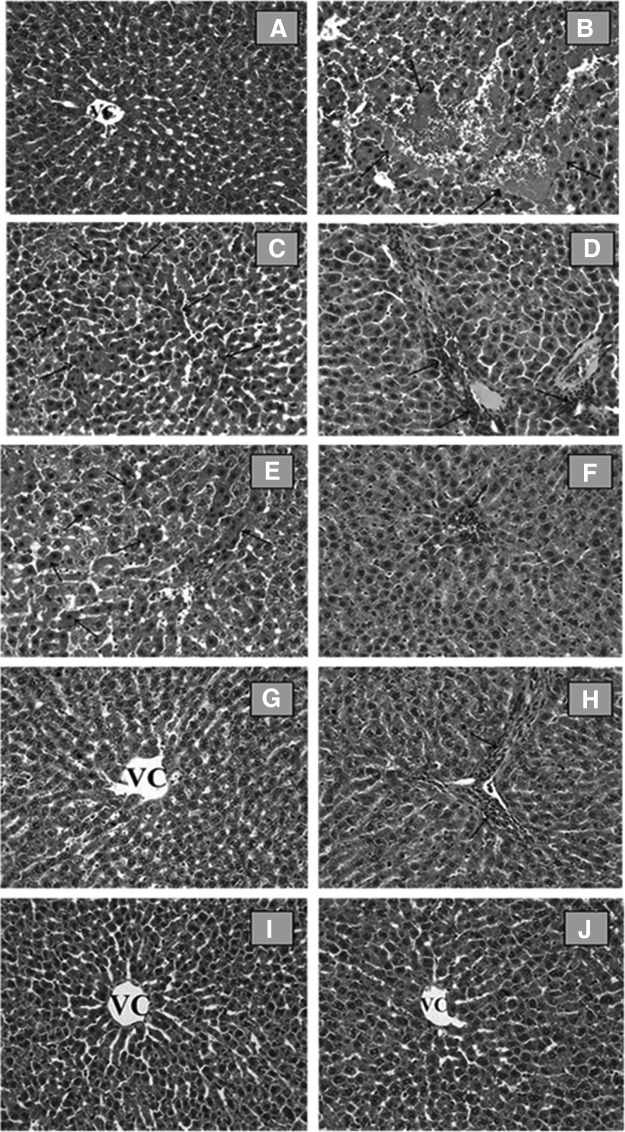
**(A)** Control group. Normal histological appearance of liver. VC: Vena centralis. H-E; × 20: **(B–D)** APAP group, **(B)** necrosis and hemorrhage (*arrows*), **(C)** eosinophilic stained and pyknotic nuclei hepatocytes (*arrows*), **(D)** mononuclear cell infiltration (*arrows*). H-E; × 20: APAP +125-MeOH, **(E)** decrease of eosinophilic stained and pyknotic nuclei cells (*arrows*) and mononuclear cell infiltration (*arrows*), **(F)** APAP +250-MeOH, **(G)** decrease of hemorrhage and mononuclear cell infiltration (*arrows*), **(H)** H-E; × 20: CUST 125-MeOH, **(I)** CUST250-MeOH, **(J)** groups. Liver tissue shows normal histological appearance. H-E; × 20. H-E, hematoxylin–eosin.

In the control group (Fig. 1A), and in the 125 and 250 mg Cuscuta extract applied groups (Fig. 1E–J), the liver demonstrated a normal histological appearance. In these groups, hepatocytes showed a normal histological appearance. Sinusoids and central vein were also clearly visible.

The livers in the APAP-intoxicated group (Fig. 1B–D) revealed severe histological alterations such as centrilobular necrosis and hemorrhage, eosinophilic stained and pyknotic nuclei cells, and mononuclear cell infiltration. Normal radial arrangements of hepatocytes from central vein were disrupted. Some of the hepatocytes in eosinophilic and in some of the hepatocytes cytoplasmic density are increased.

Eosinophilic hepatocytes in APAP +125MeOH and in APAP +250MeOH cuscuta groups were also observed, but these cells were not as widespread as in the APAP groups. These cells were decreased in these groups. Moreover, necrosis, hemorrhage, and mononuclear cell infiltration were decreased in APAP +125MeOH and in APAP +250MeOH groups. In contrast, the aqueous extracts (APAP +125Aq, APAP +250Aq) did not improve the histopathological changes from APAP-induced hepatotoxicity in rats.

### Determination of the major compound

**FIG. 2.**
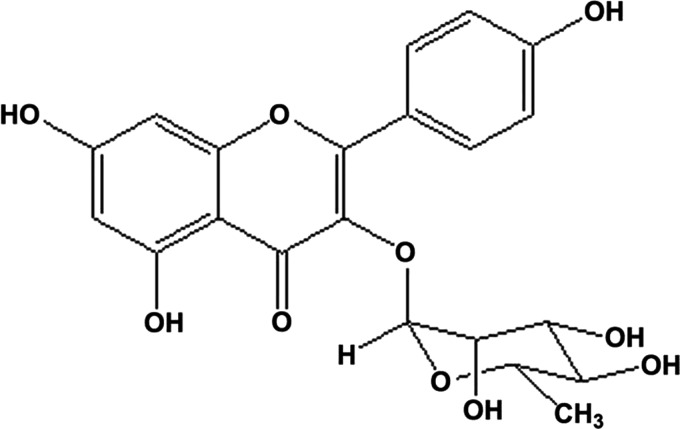
Kaempferol-3-*O*-rhamnoside.

Analysis of liver enzymes and plasma levels of biochemical parameters indicates that methanolic extracts of Cuscuta have better hepatoprotective activity than the aqueous extracts. Therefore, the methanolic extract was further fractionated and one compound was isolated and identified as 3,4′,5,7-tetrahydroxyflavone-3-*O*-rhamnoside (kaempferol-3-*O*-rhamnoside), compound **1,** on the basis of direct comparison of its spectroscopic (UV, IR, ^1^H NMR, and mass) data with those published in the literature (Fig. 2). The most abundant flavonoid in the mixture was separated (5/160 mg total fraction), with the aim of further analysis since that might be the effective compound in the raw extract.

## Discussion

This study demonstrated that the Sprague–Dawley rats (when APAP was administered intraperitoneally) were able to recover from hepatotoxic symptoms successfully. The extract significantly decreased the liver enzymes AST, ALT, and ALP levels and biochemical parameters BUN and TB in a dose-dependent manner in APAP intoxicated rats. Moreover, this extract increased antioxidant enzyme (GSH, CAT, and SOD) levels and decreased MDA levels, which is an indication of the recovery from lipid peroxidation in APAP hepatotoxicity-induced rats. Histopathological observations also confirmed that the methanolic extract-treated group prevented/recovered the histopathological changes caused by APAP intoxication such as centrilobular necrosis and infiltrating lymphocytes.

**FIG. 3.**
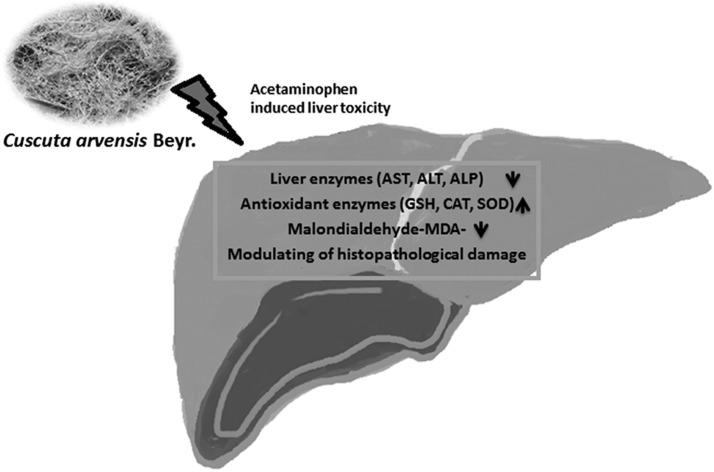
Effect mechanism of *Cuscuta arvensis*.

APAP, analgesic and antipyretic drug, is nontoxic at therapeutic doses; it is detoxified with the GSH conjugation. At toxic doses of APAP, GSH is depleted with the conjugation reaction of metabolites, in further covalent binding correlated with development of toxicity.^24^ GSH is the major nonenzymatic antioxidant and regulator of intracellular redox homeostasis in all cell types.^25,26^ GSH depletion can increase the formation of reactive oxygen species (ROS) and reactive nitrogen species (RNS) such as superoxide anion, hydroxyl radical, and hydrogen peroxide and nitro oxide. Additional levels of ROS and RNS can attack biological molecules such as DNA, protein, which leads to lipid peroxidation, and depletion of the antioxidant enzymes that later cause severe oxidative stress, DNA strand breaks, apoptosis, and necrosis.^27–29^ The measurements of GSH were in high levels when the extracts were given in both APAP-intoxicated rats and also solely extracts given rats. The mechanism of hepatoprotection by *C. arvensis* might be due to restoration of GSH. SOD catalyzes the dismutation of superoxide anion to H_2_O_2_ and O_2_; in further CAT and GPx, there is catalysis and the decomposition of H_2_O_2_ to water.^30^ Thus, the coordinate actions of various antioxidants are critical for effectively scavenging free radicals in mammalian cells. Hepatotoxic chemicals reduce the antioxidant capacity of the liver by decreasing the activity of the antioxidant enzymes. However, Cuscuta extracts prevent the reduction of antioxidant enzymes, which consequently damage the liver. Moreover, the methanolic extract of *C. arvensis* improved liver function by decreasing the serum ALT, AST, and ALP levels in hepatotoxic rats. AST is mostly related to heart and ALT is more specific to liver; therefore, in hepatotoxication, the ALT level is expected to be increased (Fig. 3). In addition, TB, which is a byproduct of the breakdown of red blood cells in the liver, is also an indicator of the liver function. Elevation of bilirubin levels reveals damage to the liver and bile duct that cause jaundice. Methanolic extract of *C. arvensis* (APAP+MeOH) declined TB levels in treated rats, indicating that it has not only a hepatoprotective effect but also enhances the liver functional efficiency. That result also suggests scientific evidence for, and confirms the folkloric usage of *C. arvensis*, which is given to the mother as well as baby (just a few drops/day) by infusion or maceration to alleviate jaundice in the newborn. A previous study on hepatoprotective effect of ethanolic *C. chinensis* extract in APAP-induced hepatoxicity showed increase in antioxidant enzyme's activities (SOD, CAT, and GPx), which is in line with our results.^31^ When the only extract given experiment groups were compared with the control group, the extract group had no negative effect on the kidney and liver and did not show toxicity overall in animals. Kaempferol 3-*O*-rutinoside and kaempferol 3-*O*-glucoside are known to have liver protective effects.^32^ A further exploration of whether the bioactive molecule kaempferol-3-*O*-rhamnoside (kaempferol glycosides derivative from flavonoids) responsible for the activity is under investigation in our laboratory. Since plant-originated pharmaceutical products especially for liver protection are limited in the market, demand of phytotherapeutic products supports further investigation on Cuscuta species.
